# Association mapping in bambara groundnut [*Vigna subterranea* (L.) Verdc.] reveals loci associated with agro-morphological traits

**DOI:** 10.1186/s12864-023-09684-9

**Published:** 2023-10-06

**Authors:** Charles U. Uba, Happiness O. Oselebe, Abush A. Tesfaye, Wosene G. Abtew

**Affiliations:** 1 Department of Horticulture and Plant Science, Jimma University, Jimma, Ethiopia; 2 Ebonyi State University Abakalilki, Abakalilki, Nigeria; 3 International Institute of Tropical Agriculture, Ibadan, Nigeria

**Keywords:** Bambara groundnut, Candidate genes, DArT SNP, Linkage disequilibrium decay, Loci, GWAS, Marker trait association

## Abstract

**Background:**

Genome-wide association studies (GWAS) are important for the acceleration of crop improvement through knowledge of marker-trait association (MTA). This report used DArT SNP markers to successfully perform GWAS on agro-morphological traits using 270 bambara groundnut [*Vigna subterranea* (L.) Verdc.] landraces sourced from diverse origins. The study aimed to identify marker traits association for nine agronomic traits using GWAS and their candidate genes. The experiment was conducted at two different locations laid out in alpha lattice design. The cowpea [*Vigna unguiculata* (L.) Walp.] reference genome (i.e. legume genome most closely related to bambara groundnut) assisted in the identification of candidate genes.

**Results:**

The analyses showed that linkage disequilibrium was found to decay rapidly with an average genetic distance of 148 kb. The broadsense heritability was relatively high and ranged from 48.39% (terminal leaf length) to 79.39% (number of pods per plant). The GWAS identified a total of 27 significant marker-trait associations (MTAs) for the nine studied traits explaining 5.27% to 24.86% of phenotypic variations. Among studied traits, the highest number of MTAs was obtained from seed coat colour (6) followed by days to flowering (5), while the least is days to maturity (1), explaining 5.76% to 11.03%, 14.5% to 19.49%, and 11.66% phenotypic variations, respectively. Also, a total of 17 candidate genes were identified, varying in number for different traits; seed coat colour (6), days to flowering (3), terminal leaf length (2), terminal leaf width (2), number of seed per pod (2), pod width (1) and days to maturity (1).

**Conclusion:**

These results revealed the prospect of GWAS in identification of SNP variations associated with agronomic traits in bambara groundnut. Also, its present new opportunity to explore GWAS and marker assisted strategies in breeding of bambara groundnut for acceleration of the crop improvement.

**Supplementary Information:**

The online version contains supplementary material available at 10.1186/s12864-023-09684-9.

## Introduction

Bambara groundnut [*Vigna subterrenea* (L) Verdc.] is a legume of sub-Sahara African origin that plays an important socio-economic role in semi-arid regions of the continent [1]. The seed is a complete food because it contains 63% carbohydrate, 19% protein, and 6.5% fat [2] and could serve as alternative in areas where animal protein is limited because of high cost [3]. Also, it could serve as herbal medicine, animal feed, green fertilizer and bio-pesticide [4, 5]. The only source of plant materials for the crop is landraces which evolved from the wild relatives [6]. Most of the landraces are poor yielders [7] and there is a need for coordinated breeding efforts to improve their productivity. There is a wide repertoire of genetic diversity and prospects for genetic improvement of bambara groundnut using an integrative and comprehensive approach that combines multi-omics approaches [8]. Paliwal et al. [9] conducted a preliminary analysis on bambara groundnut and revealed significant variation in all the accessions. Bambara groundnut is a self-pollinating crop with low level of heterozygosity among landraces and selection based on single seed descent could results to pure line [10]. Pure line selection could be used for bambara groundnut improvement because artificial hybridization is difficult [11] but the breeding process is lengthy or time consuming. Therefore, the application of molecular markers with relevant agronomic traits would significantly reduce the cost and time of developing new varieties because they accelerate the rate of genetic gain in breeding program and assist in the selection of the best parents [12].

The usage of molecular markers has aided researchers to track segments of the genome which are linked to specific phenotypes of interest in QTL-mapping and genome-wide association studies [13]. Association mapping through genome wide association study gives insight on the genetic basis of complex traits in plants and has been used in many crop species to find QTLs and candidate genes [14]. It examines associations between phenotypic variations and nucleotide polymorphism [15]. Association mapping using natural a population would study many genotypes at once and generates more precise QTL positions if a sufficient number of molecular markers are available [16]. GWAS enables the understanding of genetic architecture of phenotypic traits and shows the genetic mechanism which controls the variation of phenotypic traits [17]. Furthermore, it uses the linkage disequilibrium (LD) concept which is the non-random co-segregation of alleles at multiple loci, to survey genomic regions that describe significant variation to phenotypes [18]. The genome wide association analysis depends on markers-traits association (MTA) using genetically diverse populations and representative markers [19] with identification of MTA as the initial step for marker assisted selection and is a vital tool used in varietal improvement and rate of genetic gain [18, 20, 21]. They aid in a better understanding of genetic bases and dissection of genes which controls agro-morphological traits.

Despite the importance of GWAS in crop improvement [22] and several benefits (nutritional and economic) of bambara groundnut, its production in areas of the cultivation is still low because of the unavailability of improved varieties [23]. This has made the prospect of the crop at commercial or international level low and leads the production restricted to subsistence farmers or women in the rural areas. More so, the renewed interest in orphan crops like bambara groundnut call for actions towards creating high yielding, drought resistant, disease resistant and high nutritional value genotypes with application of molecular techniques. Unfortunately, the genome-wide association studies have not been reported in bambara groundnut to unravel the projection and variation sources linked to traits of agronomic importance or yield components [24]. Only a few reports are available on linkage-based QTL mapping for some agronomic traits using closely related crops in bambara groundnut that, suffers from poor mapping resolution, less allele mining due to the utilization of biparental population and difficult to develop mapping population. LD decay based on SNP molecular markers have been used in different crops like legumes [25, 26] and cereals [27, 28] but it has not been used in bambara groundnut. The understanding of LD decay in a population enables researcher to know the number of markers needed for association analysis [29].

Next-generation sequencing-based genotyping such as genotyping by sequencing (GBS) and diversity arrays technology-based sequencing (DArTseq) platforms have significantly contributed to the popularity of single-nucleotide polymorphisms (SNPs) [30] and enable exploration of the genetic basis of agro-morphological traits at a finer resolution. Many molecular markers have been established and used for bambara groundnut landraces for assessing the breeding system [31], diversity and population structure [11, 32] and linkage analysis [33, 34]. A linkage map using a closely related crop has been used in bambara groundnut to facilitates the identification of important gene-containing regions for some agronomic traits which could aid in marker-assisted selection (MAS) in the crop breeding programmes [34]. Association mapping has been widely used for dissecting the genetic architecture of agronomic traits in several crops: cowpea [35], sorghum [36], soybean [37], and maize [38]. Draft sequence of bambara groundnut genome has been released [2] with efforts still on going in order to assemble the complete genome of the crop. However, considering that complete sequence assembly of cowpea is available and is close relative of the bambara groundnut (both belongs to *leguminous* family and genus *Vigna*), it becomes an important tool for the analysis of regions of interest in bambara groundnut showing their high degree of collinearity, 2n = 22 [39]. Amkul et al. [40] have used similar method with cowpea reference genome to identify candidate genes controlling major QTLs in zombi pea [*Vigna vexillata* (L.) A. Rich] a legume species. This study was initiated with the objective of identifying marker traits association in bambara groundnut for some important agronomic traits using GWAS and investigate candidate genes.

## Results

### SNP analysis

**Fig. 1:**
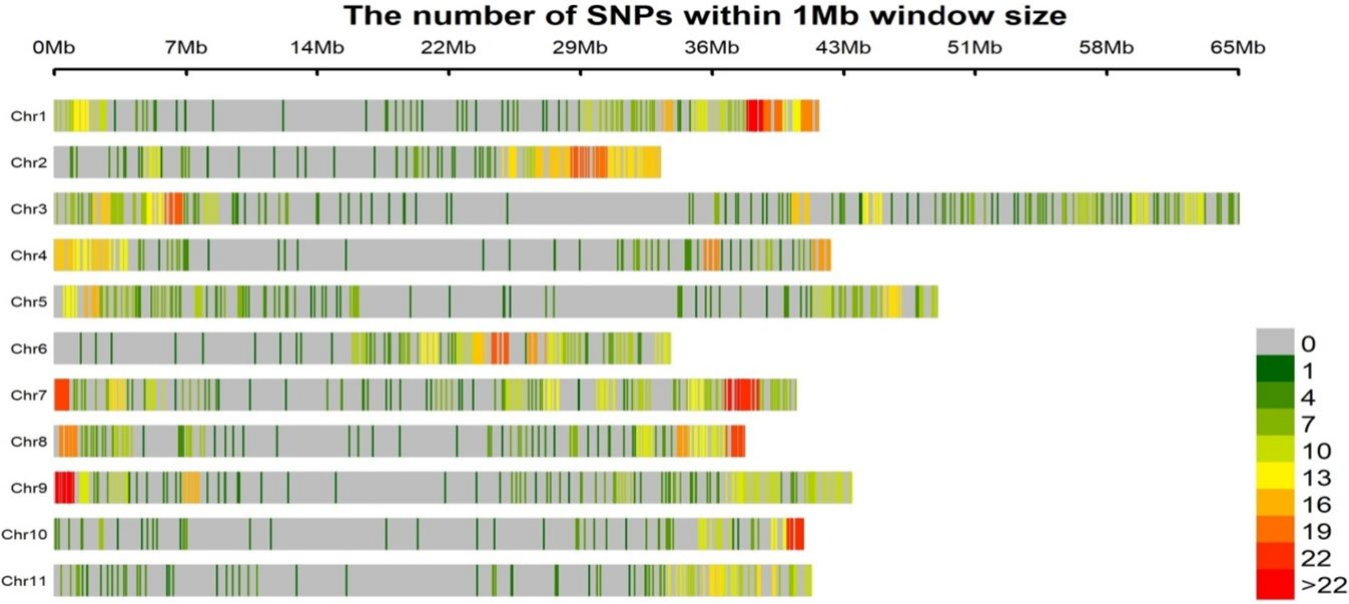
Stacked bar chart describing the density of SNPs on the chromosome. The x-axis shows the interval distance in Mb

The distribution of SNPs across the eleven chromosomes after filtering and removing of > 20% missing data and SNP that was unable to be mapped (scaffolds) to chromosome region (Table 1). The number of SNPs per chromosome varied across the eleven chromosomes. The length of individual chromosome varied from 65 Mb on chromosome 3 for highest length while chromosome 2 (33 Mb) had the lowest (Fig. 1). The marker density is not equally distributed within and between the chromosomes. The proportion of nucleotide substitutions as either transition (T/C and A/G) and transversion (G/T, A/C, G/C and A/T) are shown in supplementary Table 1. The percentage of allelic sites is T/C (30.23%), A/G (29.57%), G/T (9%), G/C (9%) and A/T (12.18%).

**Table 1:** Genomic distribution of DArT SNPs physically mapped on each chromosome used for the analysis

Chromosome number	Number of SNP	Chromosome length (Mb)	Percentage of SNP (%)	Average SNP density (kb/SNP)
1	226	42.03	10.71	186.00
2	194	33.36	9.19	171.96
3	281	65.20	13.32	232.01
4	164	42.67	7.77	260.17
5	193	48.57	9.15	251.66
6	171	33.91	8.10	198.32
7	246	40.80	11.66	165.86
8	169	37.97	8.01	224.69
9	204	43.84	9.67	214.89
10	116	41.20	5.50	355.20
11	146	41.64	6.92	285.18
Total	2110	471.19	100	2545.94

### Phenotypic variation

The analysis of variance indicated significant differences (*p* < 0.001) among the landrace for all the studied traits (Table 2). Furthermore, landrace x Location (L x Loc) interaction for most of the agro-morphological traits were highly significant except number of seeds per pod while Rep(Loc) was not significant in all the studied traits. The broad sense heritability among the traits varied from 48.39% for terminal leaf length to number of pods per plant (79.39%). All studied trait, recorded high heritability (> 60) with exception of pod length and terminal leaf length with moderate heritability.

**Table 2:** ANOVA and broad sense heritability of the measured quantitative traits used for the association mapping

Traits	Rep(Loc)	Location	Landrace	Loc x Landrace	Residuals	H_b_^2^
	df = 3	df = 2	df = 269	df = 538	df = 780	
Days to flowering (day)	27.97 ns	9272.06***	22.23***	19.55***	6.76	61.13
Days to maturity (day)	185.42 ns	38,146.92***	406.79***	192.45***	41.32	78.61
Terminal leaf length (mm)	503.06 ns	27,010.64***	192.87***	103.37***	50.65	48.39
Terminal leaf width (mm)	10.62 ns	499.80**	52.34***	20.96***	9.11	61.24
Number of pod per plant	42.81 ns	6994.46***	342.77***	116.27***	19.32	79.39
Number of seed per pod	0.03 ns	1.46 ns	0.14***	0.12 ns	0.01	65.76
Pod length (mm)	104.50 ns	4290.45***	71.33***	39.14***	22.76	54.62
Pod width (mm)	3.98 ns	13.66***	51.23***	47.30***	1.26	68.51

where *Loc* = Location, *Rep* = Replication, *H*_*b*_^*2*^ = Heritability in broadsence, *ns* = not significant

** = significant at (*p* < 0.01)

*** = significant at (*p* < 0.001)

### Correlation analysis

**Fig. 2:**
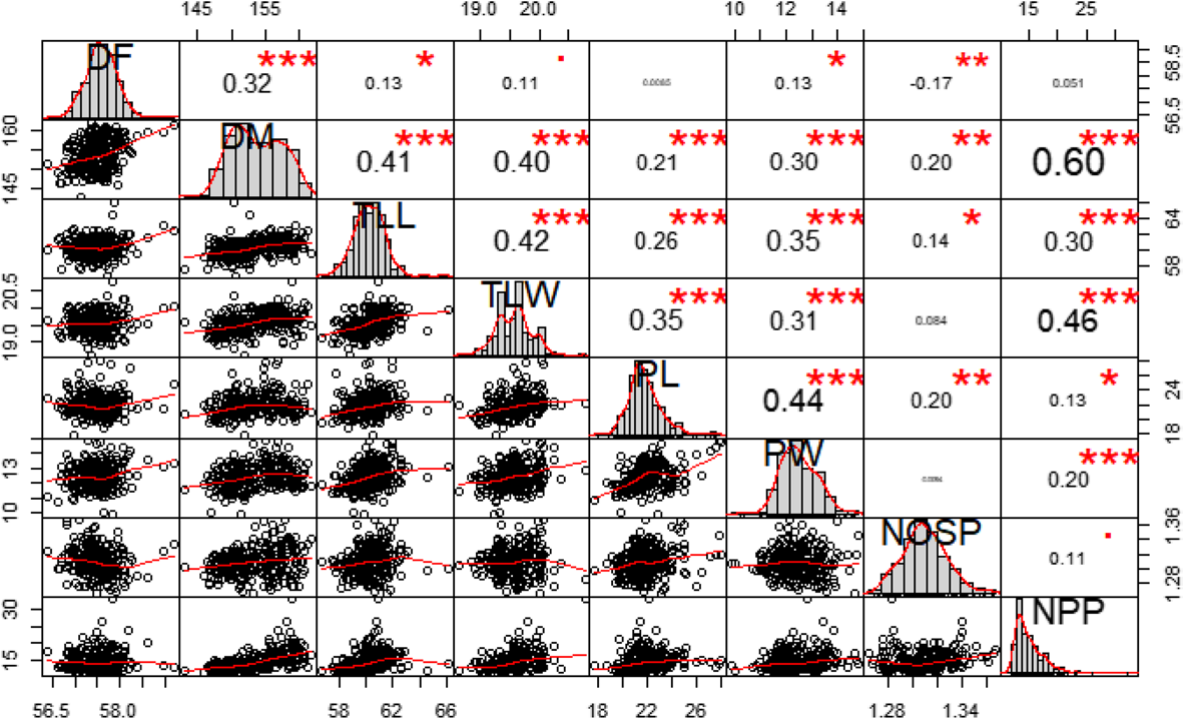
Correlation coefficient using mixed correlogram based on studied agro-morphological traits of the bambara groundnut. *DF* Days to flowering, *DM* Days to maturity, *TLL* Terminal leaflet length, *TLW* Terminal leaf width, *NPP* Number of pods per plant, *NOSP* Number of seed per pod, *PL* Pod length, *PW* Pod width

The relationship among the studied agro-morphological traits (Fig. 2) revealed strongest correlation between days to maturity and number of pods per plant (*r* = 0.60). The number of pods per plant had significant positive correlation with terminal leaf length (*r* = 0.30), terminal leaf width (*r* = 0.46), pod length (*r* = 0.13), pod width (*r* = 0.20) and number of seeds per pod (*r *= 0.11). Also, days to 50% flowering had significant correlation with days to maturity (*r* = 0.32), terminal leaf length (*r* = 0.13), terminal leaf width (*r *= 0.11), pod width (*r* = -0.13) and number of seed per pod (*r* = -0.17) while days to maturity showed significant correlation with terminal leaf length (*r* = 0.41), terminal leaf width (*r* = 0.40), pod length (*r* = 0.21), number of seeds per pod (*r* = 0.20) and number pods per plant (*r* = 0.60).

### LD analysis

**Fig. 3:**
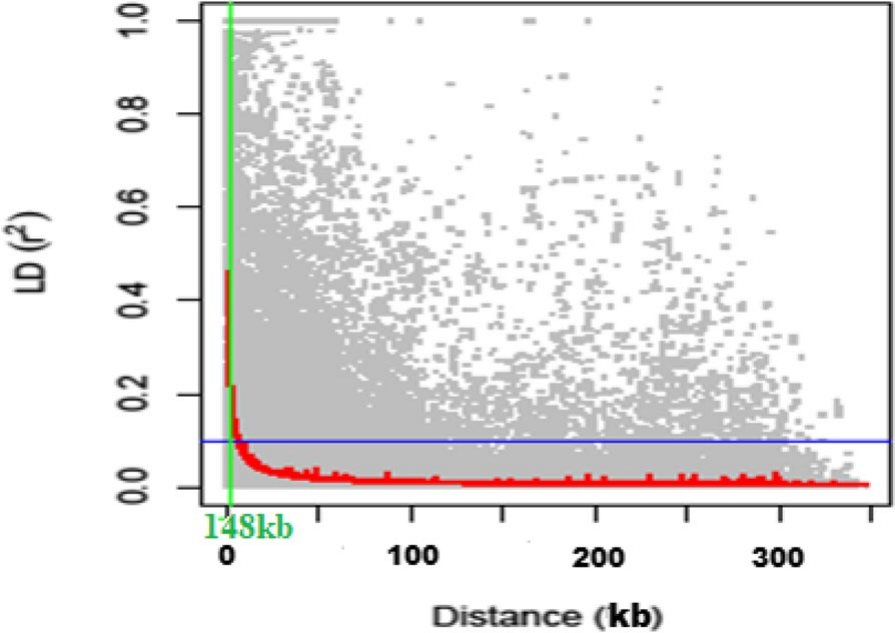
Linkage disequilibrium decay in the bambara groundnut germplasm

The distribution of pairwise LD values (r^2^) associated with all the chromosomes were determined based on the genotyping information for 2110 genome wide SNPs in the 270 bambara groundnut landraces. A total of 104,226 intra-chromosomal pairs were generated from the LD analysis and the mean r^2^ of all pairs is 0.1 (Supplementary Table 2). The number of r^2^ significant (*p* < 0.005) pairs was 21,852 (20.97%) and a mean of 0.23. A non-linear regression curve exhibiting a decreasing trend of LD decay with an increase in the physical distance was observed (Fig. 3). The graph had a red curve line, which is the smoothing spline regression model fitted to LD decay, and a blue horizontal straight-line indicating the threshold above which r^2^ values are likely due to genetic linkage. The analysis yielded an average linkage disequilibrium decay of 148 kb and a whole genome average maximum r^2^ of 0.46.

### Genome-wide marker-trait associations and candidate genes

**Fig. 4:**
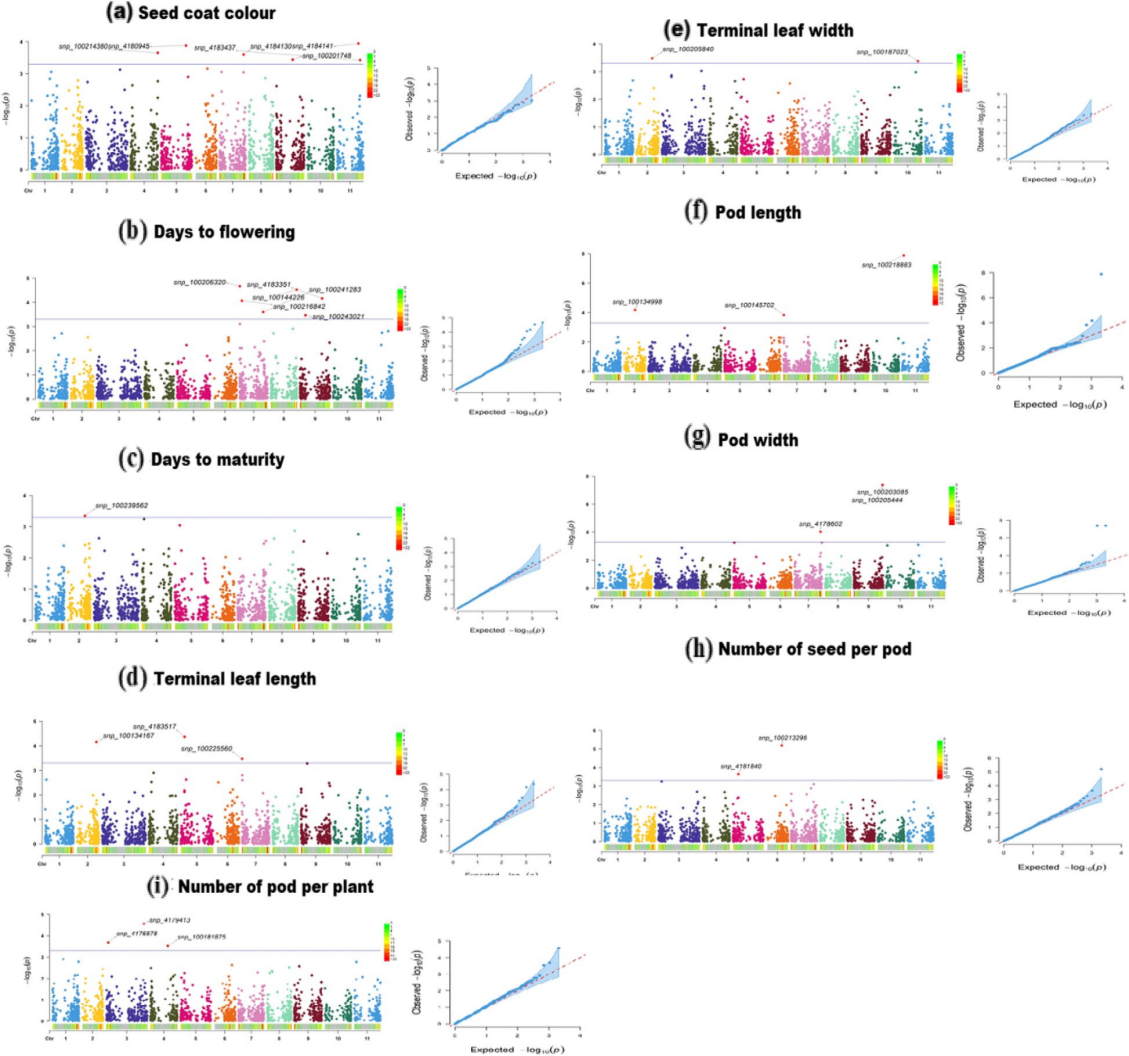
Manhattan plot of GWAS for the nine studied agronomic traits. The y axis refers to -log10 (p) values plotted against physical position on each of the 11 chromosomes. Dashed line indicates genome-wide significance threshold

The maker trait associations (MTAs) identified a total of 28 significant SNPs for the studied agro-morphological traits in the bambara groundnut population and were distributed across nine of the eleven chromosomes (Table 3, Fig. 4). The MTA identified for each trait varied from 6 markers (seed coat colour and days to flowering) to 1 marker for days to maturity. Similarly, the number of significant SNPs identified on each chromosome ranged from 1 (chromosome 8 and 10) to 6 (chromosome 7) while no marker trait association was identified on chromosome 1. The percentage of phenotypic variation for identified significant SNP markers ranged from 5.27% for number of pods per plant to 24.86% for pod width. A total of 17 candidates’ genes were identified among all the nine traits studied (Table 4). The present study identified candidate gene for seed coat colour (6), days to flowering (3), days to maturity (1), terminal leaf length (2), terminal leaf width (2), pod width (1) and number of seed per pod (2).

**Table 3:** Significant marker-trait associations identified in the GWAS analysis

Traits	SNP	Chr	Position	Allele	SNP Effect	*P* value	PVE (%)
Seed coat colour	snp_4184141	10	33,814,471	C/A	1.74	0.000113	11.03
	snp_4180945	5	39,211,932	C/G	1.524	0.000132	5.76
	snp_100214380	4	42,537,063	C/T	-1.05	0.000223	8.25
	snp_4184130	7	37,129,987	G/A	0.72	0.000251	8.99
	snp_100201748	9	25,647,130	T/C	0.81	0.000361	10.94
	snp_4183437	11	36,459,384	A/T	0.69	0.000375	9.06
Days to flowering	snp_100206320	7	138,798	T/A	2.24	2.17E-05	14.87
	snp_100216842	7	3,200,663	G/C	3.02	8.59E-05	15.82
	snp_100241283	7	34,380,456	C/T	1.26	0.00025	17.03
	snp_4183351	9	32,254,073	G/C	2.12	6.86E-05	14.58
	snp_100243021	9	7,898,627	C/T	-1.34	0.00034	19.49
	snp_100144226	8	37,729,441	C/T	2.45	3E-05	14.53
Days to pod maturity	snp_100239562	2	25,192,034	T/C	7.56	0.00045	11.66
Terminal leaf length	snp_4183517	5	6,059,547	C/T	-2.85	4.31E-05	9.04
	snp_100134167	2	29,673,801	T/G	6.41	6.94E-05	13.86
	snp_100225560	7	173,610	C/T	4.08	0.000339	9.43
Terminal leaf width	snp_100205840	3	23,864,310	G/A	2.36	0.00033	10.16
	snp_100187023	10	35,780,968	C/T	3.01	0.000418	6.82
Pod length	snp_100218883	11	2,917,453	T/C	8.12	1.3E-08	24.42
	snp_100134998	2	17,642,501	A/C	3.35	6.71E-05	12.77
Pod width	snp_100203085	9	42,837,031	C/T	-2.01	4.21E-08	24.86
	snp_100205444	9	43,175,973	G/T	-2.01	4.21E-08	17.79
	snp_4178602	7	38,442,799	C/G	-1.68	9.5E-05	10.68
Number of seed per pod	snp_100213296	6	24,122,517	T/C	0.06	6.47E-06	11.58
	snp_4181840	5	8,927,972	G/T	-0.03	0.000229	11.47
Number of pods per plant	snp_4179413	3	59,879,317	C/A	-5.80	2.72E-05	5.27
	snp_4176878	3	3,393,003	G/A	5.02	0.000205	7.33
	snp_100181875	4	27,546,978	A/G	-4.20	0.0003	11.49

*Chr*. Chromosome, *PVE* Phenotypic variance explained

**Table 4:** Candidate genes related to some agronomic traits identified

Trait	Locus name	Chr	SNP	Start	End	Gene name	Species	Reference
SCC	Vigun10g129300	10	snp_4184141	33,732,100	33,726,737	MYC // Transcription factor MYC1	Wheat	[61]
	Vigun10g129800	10	snp_4184141	33,807,377	33,790,615	F-box/kelch-repeat protein	Wheat	[62]
	Vigun05g203400	5	snp_4180945	39,196,947	39,189,868	Kinesin-2-related	Groundnut	[63]
	Vigun07g253400	7	snp_4184130	37,214,868	37,213,449	2OG-FE II Oxygenase	Rapeseed	[64]
	Vigun09g116600	9	snp_100201748	25,555,298	25,552,437	2OG-FE II Oxygenase	Rapeseed	[64]
	Vigun11g157200	11	snp_4183437	36,595,286	36,590,650	E3 ubiquitin-protein ligase	Cowpea	
DF	Vigun07g034000	7	snp_100216842	3,256,536	3,252,390	MADS box protein // Agamous-like MADS-box protein AGL18	Arabidopsis	[66]
	Vigun09g157400	9	snp_4183351	32,340,826	32,336,700	F-box protein—FKF1	Soybean	[67]
	Vigun09g071700	9	snp_100243021	7,868,711	7,865,812	C2H2-type zinc finger (zf-C2H2_6)		[69]
DPM	Vigun02g095500	2	snp_100239562	25,121,565	25,109,818	ATS1 genes	Arabidopsis	[70]
TLL	Vigun02g150200	2	snp_100134167	29,719,503	29,716,096	BTB/POZ domain proteins	Arabidopsis	[71]
	Vigun02g150500	2	snp_100134167	29,743,941	29,741,874	LOB domain-containing protein 37-related	Maize	[73]
TLW	Vigun02g085200	3	snp_100205840	23,962,357	23,950,835	Protein kinase domain (Pkinase)	Arabidopsis	[72]
	Vigun10g140300	10	snp_100187023	35,743,746	35,739,978	BTB/POZ domain (BTB)	Arabidopsis	[71]
NOSP	Vigun06g111300	6	snp_100213296	24,033,017	24,026,490	Serine/Threonine-protein kinase	Maize	
	Vigun06g111600	6	snp_100213296	24,053,108	24,051,150	B3 DNA binding domain (B3)	Zombi pea	[40]
PW	Vigun09g269300	9	snp_100205444	43,212,110	43,207,233	Zinc finger CCHC domain containing protein	*Medicago truncatula*	[77]

*SCC* Seed coat colour, *DF* Days to flowering, *DM* Days to maturity, *TLL* Terminal leaf length, *TLW* Terminal leaf width, *NOSP* Number of seed per pod, *PW* Pod width, *Chr* Chromosome

The MTAs on seed coat colour trait identified six candidate genes controlling seed pigment and seed coat colour (Vigun10g129300, Vigun10g129800, Vigun05g203400, Vigun07g253400, Vigun09g116600 and Vigun11g157200) and the percentage of phenotypic variation among the significant SNP markers varied from 5.76% to 11.03%. However, on days to flowering three candidate genes (Vigun07g034000, Vigun09g157400 and Vigun09g071700) that regulate flowering time was identified with the percentage of phenotypic variation among significant markers ranges from 14.53% to 19.49% while for days to maturity only one candidate gene (Vigun02g095500) responsible for pod maturity was identified and the significant marker explained 11.66% of the phenotypic variation. Furthermore, candidate genes responsible for terminal leaf length (Vigun02g150200 and Vigun02g150500) and width (Vigun02g085200 and Vigun10g140300) which is one of the key organs for photosynthesis were identified. Pod length and width was analyzed as a measure of increase in organ size with one candidate gene for pod width (Vigun09g269300) identified. In addition, candidate gene identified for number of seed per pod (Vigun06g111300 and Vigun06g111300) trait which play significant importance on bambara groundnut yield.

## Discussion

### SNP density

The marker density revealed that SNPs are in abundance in telemetric regions of the chromosome than the pericentrometric regions and they varied across chromosome arm positions. This could be due to bias from restriction enzymes used or difference in gene coverage. Similar findings have been reported by Serba et al. [28]. Furthermore, frequency of SNPs showed higher transitions substitution (59.8%) than tranversion substitution (40.37%). This agrees with previous report on genome-wide SNP in other crops [41–43]. The higher frequent of transitions substitution indicates they are better tolerated during natural selection because they are synonymous mutations in protein-coding sequences [44].

### Linkage disequilibrium in bambara groundnut

This is the first report of quantification of LD decay in bambara groundnut, defining the extent of LD in the germplasm. The extent of genome-wide recombination is expected to be lower in self-pollinated crop like bambara groundnut than what is observed in cross pollinated crops [45]. A rapid LD decay of 148 kb was observed in the 270 bambara groundnut landraces. This information is helpful for bambara groundnut breeders when considering the population composition and selection of markers for further association mapping studies. Thus, the rapid LD decay suggests that a small number of markers could provide sufficient genome coverage for finding marker-traits associations with a diverse bambara groundnut population, although having higher number of markers are more desirable because it would lead to identification of more MTAs. Jia et al. [46] noted that rapid LD decay revealed that the population used are sufficiently diversified and are suitable for GWAS analysis. Furthermore, the rapid LD decay observed could be that bambara groundnut landraces have undergone more recombination and had higher genetic diversity. Higher genetic diversity of bambara groundnut landraces has been previously reported by Uba et al. [32]. These LD decay estimates obtained in this bambara groundnut study are higher than previously published values for other self-pollinated crops like *Medicago truncatula*: 3 kb [47], *Arabidopsis thaliana*: 3–4 kb [48], sorghum: 50–100 kb [49], foxtail millet: 100 kb [46], cowpea: 80–100 kb [26], cultivated mungbean: 100 kb [50] and *Oryza sativa* Indica: 123 kb [51] while is similar with cultivated soybean: 150 kb [52]. However, the LD decay values are lower than previously reported on *Oryza sativa* Japonica: 167 kb [51], common bean: 400 kb [53] and chickpea: 450–550 kb [54] for self-pollinated crops.

### Genome-wide association mapping

In bambara groundnut, previous report aimed at elucidating the genetics of agronomic traits have mainly focused on the maker traits association using biparental cross. This few available report on biparental cross used genetic linkage groups and was not identified based on bambara groundnut or cowpea reference genomes. Hence, it could not be compared directly with our results because there is no available literature. Understanding the diversity and population structure is in a germplasm panel is importance prerequisite needed for GWAS and has been performed in our previous report [32] and it indicates that the panel used are diverse. This study has successfully demonstrated that GWAS can be used in bambara groundnut to identify candidate genes and that GWAS could be used to identify important agronomic traits in the crop with reduced cost and time. The already available cowpea reference genome sequence [55] and functional annotation made it possible for us to identify putative candidate genes for some traits in bambara groundnut. The heritability estimates for most of studied traits were generally high and agreed with previous studies on bambara groundnut [56, 57]. Efforts in locating genes influencing various agronomic traits will aid to facilitate marker assisted selection application in bambara groundnut. In this study, some significant MTAs have been found on chromosomes previously reported to contain QTL for some traits in cowpea. Example, seed coat colour was reported to be correlated with genomic regions on chromosomes 7, 9 and 10 [58] while on flowering time for chromosome 9 [59]. These identified candidate genes were co-located with known or functionally related genes.

Seed coat colour is an important trait used for the characterization of bambara groundnut [60] and affects consumers’ preferences. These candidates’ genes on seed coat colour that were identified on chromosome 5, 7, 9, 10 and 11 revealed that these chromosomes are very important in controlling this trait in bambara groundnut. Herniter et al. [58] has pointed out the important of these chromosomes (5, 7, 9 and 10) in controlling seed coat colour in cowpea. Our data identified these candidate genes (Vigun10g129300, Vigun10g129800, Vigun05g203400, Vigun07g253400, Vigun09g116600 and Vigun11g157200) for seed coat colour. The gene Vigun10g129300 (MYC1 transcription) encodes for TaMYCI involved in seed pericarp colour of wheat [61] while Vigun10g129800 gene (F-box/kelch-repeat protein) plays important role in seed pigmentation in wheat [62]. The Vigun05g203400 gene (Kinesin-2-related protein) is responsible for seed coat cracking and brown colour in *Arachis hypogaea* L [63]. Furthermore, candidate gene Vigun07g253400 and Vigun09g116600 (2OG-FE II Oxygenase superfamily protein) are involve in seed pigment metabolism for seed coat colour in rapeseed [64] while the gene Vigun11g157200 (E3 ubiquitin-protein ligase) involves in seed coat colour for cowpea [59].

Flowering time is an important agronomic trait that plays key role in the adaptation of a variety to specific agro-ecological areas. Several significant SNPs identified on chromosome 7 and 9 in the current study have shown the important of these chromosomes in controlling flowering time and adaptation in this crop. In an earlier study in cowpea, Paudel et al. [65] obtained similar result and noted that many significant SNPs identified in a chromosome for flowering time suggests important of the chromosome on the trait. Three candidate’s genes (Vigun07g034000, Vigun09g157400 and Vigun09g071700) that regulate flowering time were identified. The Vigun07g034000 gene (MADS box protein / Agamous-like MADS-box protein AGL18) have been reported to be responsible for floral repressor that affects flowering time in Arabidopsis [66] while Vigun09g157400 gene (F-box protein, FKF1) affects flowering time in soybeans [67] and tomatoes [68]. The Vigun09g071700 gene (C2H2-type zinc finger) can modify the chromatin of FLOWERING LOCUS C (FLC) involve in the transcriptional regulation of flowering induction in Arabidopsis [69]. However, for days to maturity the gene Vigun02g095500 (ATS1) gene have been reported to have distinct developmental functions unique to the maturing embryo in Arabidopsis [70].

Leaf size (terminal leaf length and width) is an important organ responsible for photosynthesis and plays an important role during plant growth and development. Two candidate genes were identified terminal leaf length (Vigun02g150200 and Vigun02g150500) and two for terminal leaf width (Vigun02g085200 and Vigun10g140300). The gene Vigun02g150200 and Vigun02g085200 (BTB/POZ domain protein) encodes BLADE-ON-PETIOLE1 required for leaf morphogenesis in Arabidopsis thaliana [71]. In addition, Vigun02g150500 gene (protein kinase domain, Pkinase) encode Tousled gene required for leaf and flower development in Arabidopsis [72]. Similarly, Vigun10g140300 candidate gene (LOB domain transcription factors) functions to keep KNOX gene out of the initiating leaf in maize that affects leaf development [73].

The number of seeds per pod and pod size (pod length and width) plays key roles in the yield of legumes. Two candidate genes were identified for number of seeds per pod (Vigun06g111600 and Vigun06g111300) while one for pod size (Vigun09g269300). The candidate gene Vigun06g111600 (B3 DNA-binding domain protein) for number of seed per pod plays important role on development of ovule [40, 74] and reproductive meristem (REM) genes (i.e. REM34, REM35 and REM36) that influence male and female gametophyte development in Arabidopsis [75]. Furthermore, Vigun06g111300 gene (Serine/Threonine-protein kinase) encodes KNR6 gene on maize and regulates number of kernels per row with yield increase [76]. For pod width, the candidate gene Vigun09g269300 encodes Zinc finger CCHC domain containing protein in *Medicago truncatula* involve in seed size [77].

It is important to highlight the challenges faced in the present study when finding marker trait associations because of the limited number of landraces used and unavailability of the complete reference genome of bambara groundnut. The present study identified few markers trait associations but using higher number of landraces with diverse population and reference genome of the bambara groundnut would lead to detection of higher number of MTAs. The effect of population size on GWAS has been noted and the detection power of MTAs decreased according to reduction of population size [78]. Alqudah et al. [22] reported that a range of 100 to 500 genotypes are suitable for performing GWAS and the selection genotype population should consider the genetic background, genotypic and phenotypic variation. Although, population size affects power of GWAS [78], but the result of the present study should be considered early evidence about the genomic regions and markers associated with the study traits and should be further validated using higher number of genotypes in more diverse environments.

## Conclusion

This study reports marker traits association and candidate genes identified for agro-morphological traits using 270 bambara groundnut landraces diversity panel. The LD analysis revealed that the germplasm panel decayed rapidly at 148 kb. The GWAS using MLM mode identified a total of 28 MTAs for the studied agro-morphological traits with 17 candidate genes which would be beneficial in marker assisted selection and traits introgression in bambara groundnut after their validation. Overall, this finding provides new insight on the prospect or application of GWAS on bambara groundnut that would promote the use of marker assisted breeding strategies for future use in the crop.

## Material and methods

### Plant material and phenotyping

**Fig. 5:**
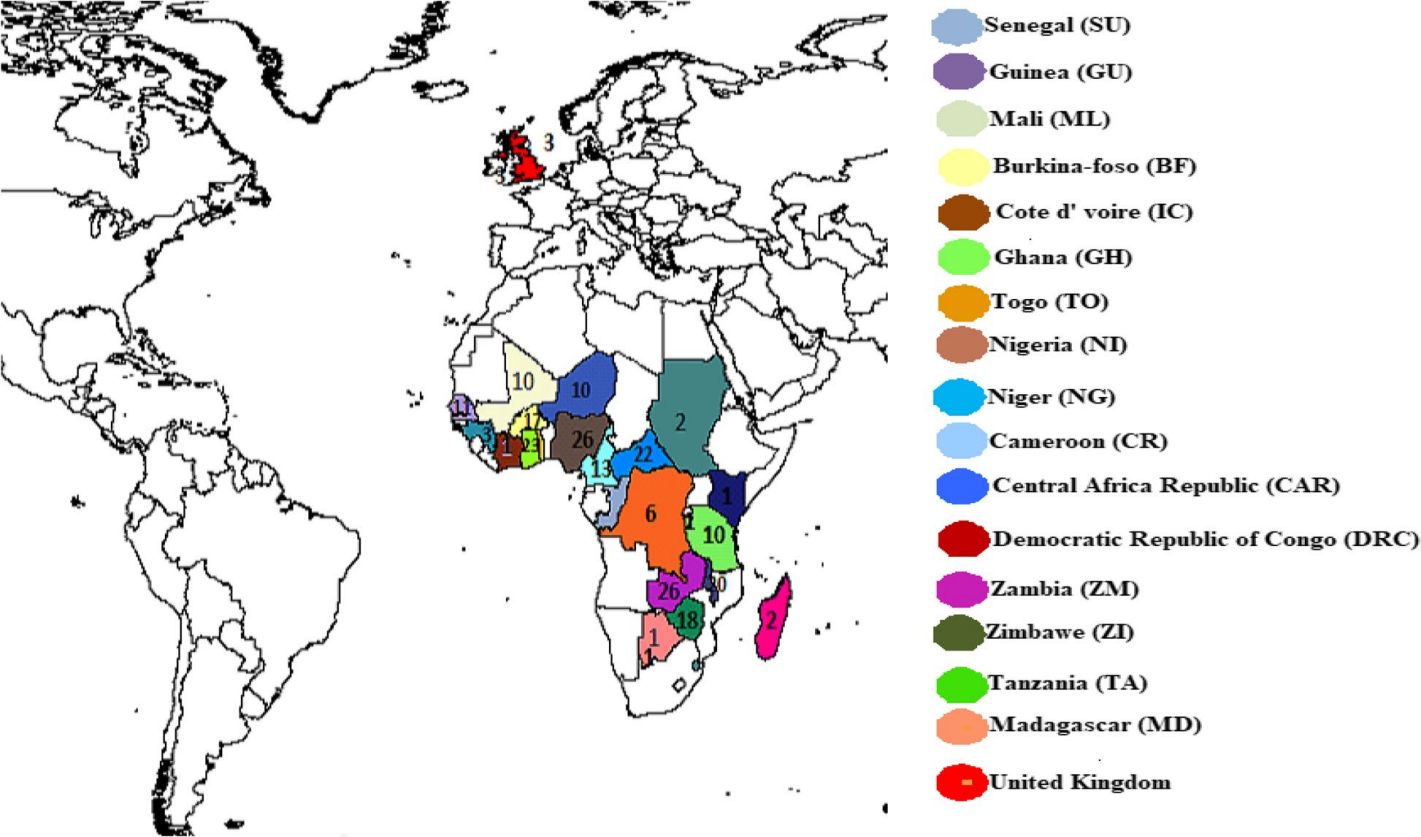
Geographical distribution of the 270 bambara groundnut accessions generated using DIVA-GIS software (version 1.4) environment http://www.diva-gis.org [80]

The study comprised a collection of 270 landraces of bambara groundnut representing 17 countries from different regions of Africa (West, South, Central and East) and unknown origin sourced from United Kingdom germplasm. The landraces were obtained from the gene bank of International Institute for Tropical Agriculture (IITA), Ibadan, Nigeria and Crop and Soil Science Research Farm in Bunda, Malawi (Fig. 5, Supplementary Table 3). The landraces were phenotyped in the year 2019 at one location and in the year 2020 at two locations in Ethiopia. The phenotyping for the year 2019 was conducted at research and experimental farm of Jimma University in Oromia Regional State (designated as Location 1) while in the year 2020 it was repeated at the same site used in the previous year (designated as Location 2) with additional site at Tepi agricultural research center (TARC) SNNP Regional State (designated as Location 3). The landraces were evaluated using an alpha lattice design with two replications. Two seeds were planted per hill at a depth of 5 cm with inter and intra row spacing of 50 cm × 30 cm, respectively, and later thinned to one seed per hill after emergence. Best management practices recommended for bambara groundnut crop management were adopted in all the locations. Nine agro-morphological traits (seed coat colour, days to flowering, days to maturity, terminal leaflet length, terminal leaf width, number of pods per plant, number of seed per pod, pod length and pod width) were selected and measured according to the descriptors established for bambara groundnut [79].

### Genotyping

Young leaves from each landrace were harvested from the greenhouse and put inside eppendorf tube with dry ice. The harvested leaf tissue was stored at − 80˚C and was lyophilized, then ground in tubes using a plate shaker. The DNA was extracted using Nucleomag Plant Genomic DNA extraction kit according to the procedure of the manufacturers and the quality of the DNA was checked. Kilian et al. [81] procedure was used to construct the library and Diversity Arrays Technology and Sequencing (DArTSeq) complexity reduction was done, through PstI-TaqI digestion of genomic DNA and ligation of barcoded adapters followed by PCR amplification of adapter-ligated fragments. It was sequenced using single read sequencing run for 77 bases using Hiseq2500. SNP markers were aligned to the completed reference genome of cowpea (*Vigna unguiculata* (L.) Walp) [55]. The markers that can be mapped to the reference genome were selected further for polymorphism between all landraces. DArTseq marker scoring was conducted using the DArT Proprietary Limited (PL’S) proprietary SNP calling algorithms (DArTsoft 14) and the SNP markers were scored as “0” = reference allele homozygote, “1” = SNP allele homozygote and “2” = heterozygote. The SNP marker quality was evaluated based on the individual marker related statistics according to Triticarte Pty Ltd. None polymorphic markers were removed and we selected SNP markers above 80% call rate and 95% reproducibility. TASSEL software [82] was employed for further filtering and SNPs with more than 20% missing values were removed.

### Statistical analysis

#### Phenotypic analysis

The analysis of phenotypic data for alpha lattice design was computed with R software [83]. A mixed model was fitted per location and across locations for each trait to estimate the best linear unbiased estimators (BLUEs) of landraces means using multi environment trial analysis with (META-R) programme [84]. The analysis of variance (ANOVA) was performed across locations for landraces, location, replication nested within location and landrace-by-location interactions.

$${P}_{ijk}=\mu +{G}_{i}+{R}_{j}+{B}_{k(j)}+{\upepsilon }_{ijk}$$

where P_ijk_ = phenotypic response, µ = grand mean, G_i_ = is the genetic effect of the genotype i, R_j_ = is the fixed replicate effect of the replicate j; B_k_(_j_) = is the effect of incomplete block k within replication j and, ɛ_ijk_ = the residual error.

The broad-sense heritability of the combined location analysis was calculated with the formula:

$${\mathrm{Hb}}^{2}=\frac{ {\sigma }_{g }^{2}}{{\sigma }_{g }^{2}+\frac{{\sigma }_{ge }^{2}}{nLoc} + \frac{ {\sigma }_{e }^{2}}{nLoc x nRep}}$$

where Hb^2^ is broad-sense heritability, ${\sigma }_{g }^{2}$ and ${\sigma }_{e }^{2}$ are the genotype and error variance components, respectively, ${\sigma }_{ge }^{2}$ is the G × L interaction variance component, nRep is the number of replicates, and nLoc is the number of locations.

Spearman’s correlation was analyzed to understand the relationship among the studied agro-morphological traits using “PerfomanceAnalytics” package [85].

### Linkage disequilibrium analysis

The Linkage disequilibrium (LD) was calculated as the squared allele frequency correlations (r^2^) using TASSEL 5.2.33 [82]. Pairwise LD r^2^ values were plotted against the corresponding physical distance, and a non-linear regression model was fitted to estimate the genome wide LD decay according to Raman et al. [86]. A value of *p* < 0.005 was considered the significance threshold for marker pairs to be in LD with each other. The LD decay was drawn using R statistical package [83] and a “LOESS” regression curve was fitted. The average LD decay of the association mapping panel was determined as the point at which LD curve intercepts the critical r^2^.

### GWAS analysis

The GWAS was conducted using mixed linear model (MLM) algorithm implemented in R package [87]. The model incorporated the principal component analysis (PCA) to account for the population structure (Q) and kinship coefficient matrix (K) to correct for relatedness of the landraces. The BLUEs values for each trait generated from the META-R analysis were taken as the phenotype and the SNP markers taken as the genotype for GWAS. The dataset for the three locations were combined using meta-analysis according to Lo et al. [88]. Meta-analysis is effective in increasing the power of association and detects genotype-by-environment interaction loci. Meta-analysis has the potential of overcoming limitations of individual environment by increasing the resolution power and reduction in false-positive findings [89]. This method has been used in a cowpea experiment that involves multiple environments [88] and loci that have different effects across different environments are G × E interaction loci. Markers that revealed significant associations were retained as true phenotype-to-genotype associations. The GWAS results were represented in a Manhattan plot using the R package “CMplot” (https://github.com/YinLiLin/R-CMplot). The significant P-values (1/n; n = total numbers of SNP markers used) for the plant genome wide association studies was computed as described by several authors [90–92] and Bonferroni correction was applied to set thresholds for controlling the genome-wide type 1 error rate. Furthermore, the model fit was tested using the quantile–quantile (QQ) plot considering the deviation of the observed test statistics values from the expected test statistics values. Each SNP percentage contribution to the phenotypic variation was estimated using marker R^2^ values multiplied by 100.

### Candidate genes analysis

The cowpea reference genome [55] was used to determine the underlying candidate genes. To identify candidate genes, local LD decay was computed using TASSEL 5.2.33 software [82] to capture flanking regions of up to 148 kb on either side of significant SNPs. The gene models together with their functional annotation were obtained from the Joint Genome Institute cowpea genome portal (www.phytozome.net). Candidates were selected based on whether the function of the genes had been characterized before in cowpea or if similar genes in other species had known roles.

### Supplementary Information

**Additional file 1.**

## Data Availability

The datasets generated during the present study are available in the submitted manuscript and supplementary information document.

## Abbreviations

ANOVA: Analysis of variance
BLUEs: Best linear unbiased estimators
DArTseq: Diversity arrays technology-based sequencing
GWAS: Genome-wide association studies
GBS: Genotyping by sequencing
IITA: International Institute for Tropical Agriculture
LD: Linkage disequilibrium
MTA: Marker trait association
MLM: Mixed linear model
PCA: Principal component analysis
QTL: Quantitative trait loci
QQ: Quantile-quantile
REM: Reproductive meristem
SNP: Single-nucleotide polymorphisms
