# Supplementary material for: Association mapping in bambara groundnut [Vigna subterranea (L.) Verdc.] reveals loci associated with agro-morphological traits
Source: BMC Genomics. 2023 Oct 6;24:593. doi: 10.1186/s12864-023-09684-9 (PMC10557193; doi:10.1186/s12864-023-09684-9)
Supplement: Supplementary file 1 — Additional file 1. [file 12864_2023_9684_MOESM1_ESM.docx]

**Supplementary Table 1:** Proportion of transition and transversion of the 2110 SNPs used for the analysis

| Allelic information | Transition | |  | Transversion | | | |
| --- | --- | --- | --- | --- | --- | --- | --- |
|  | T/C | A/G |  | G/T | A/C | G/C | A/T |
| Number of allelic sites | 638 | 624 |  | 186 | 215 | 190 | 257 |
| Percentage of allelic sites (%) | 30.23 | 29.57 |  | 9 | 10.19 | 9 | 12.18 |
| Total | 1262 | |  | 848 | | | |
| Percentage (%) | 59.8 | |  | 40.37 | | | |

**Suplementary Table 2:** Summary of linkage disequilibrium analysis in the bambara groundnut landrace population

| LD estimates | LD profile of the population |
| --- | --- |
| Total pairs of LD | 104,226 |
| Number of pairs in complete LD | 39,073 |
| Percentage of pairs in complete LD | 37.49% |
| Mean r^2^ for all pairs | 0.10 |
| Number of significant pairs | 21,852 |
| Percentage of significant pairs (%) | 20.97% |
| Mean r^2^ for all significant pairs | 0.23 |
| LD decay | 148kb |

p < 0.005

**Supplementary Table 3**

**The names of genotypes used in the study and their countries of origin**

| **S/N** | **Genotype Name** | **Country** | **S/N** | **Genotype Name** | **Country** |
| --- | --- | --- | --- | --- | --- |
| 1 | TVSu-88 | Mali | 136 | TVSu-182 | Nigeria |
| 2 | TVSu-565 | Cameroon | 137 | TVSu-1971 | Swaziland |
| 3 | TVSu-2007 | DRC | 138 | TVSu-1383 | Togo |
| 4 | MADURA AMPHETA | Malawi | 139 | TVSu-1628 | Togo |
| 5 | TVSu-235 | Ghana | 140 | TVSu-893 | Zambia |
| 6 | TVSu-305 | Burkina-Faso | 141 | TVSu-1411 | Togo |
| 7 | TVSu-412 | Cameroon | 142 | TVSu-377 | Tanzania |
| 8 | TVSu-792 | Kenya | 143 | TVSu-1860 | Zimbabwe |
| 9 | TVSu-1161 | Burkina-Faso | 144 | TVSu-226 | Ghana |
| 10 | TVSu-1381 | Unknown origin sourced from United Kingdom | 145 | TVSu-247 | Gambia |
| 11 | TVSu-1457 | Ghana | 146 | TVSu-297 | Burkina-Faso |
| 12 | TVSu-1797 | Malawi | 147 | TVSu-1449 | Ghana |
| 13 | TVSu-93 | Mali | 148 | TVSu-1832 | Niger |
| 14 | TVSu-1844 | Niger | 149 | TVSu-1981 | DRC |
| 15 | TVSu-1610 | Togo | 150 | TVSu-138 | Ghana |
| 16 | TVSu-358 | Gambia | 151 | TVSu-1630 | Togo |
| 17 | TVSu-460 | Cameroon | 152 | TVSu-877 | Zambia |
| 18 | TVSu-810 | Madagascar | 153 | TVSu-1405 | Togo |
| 19 | TVSu-2008 | DRC | 154 | TVSu-378 | Tanzania |
| 20 | TVSu-1364 | CAR | 155 | TVSu-186 | Benin |
| 21 | TVSu-1668 | Senegal | 156 | TVSu-236 | Ghana |
| 22 | TVSu-1799 | Malawi | 157 | TVSu-248 | Gambia |
| 23 | TVSu-100 | Mali | 158 | TVSu-294 | Burkina-Faso |
| 24 | TVSu-1887 | Tanzania | 159 | TVSu-1453 | Ghana |
| 25 | KAYERA | Malawi | 160 | TVSu-1834 | Niger |
| 26 | TVSu-1607 | Togo | 161 | TVSu-200 | Benin |
| 27 | TVSu-345 | Gambia | 162 | TVSu-1363 | CAR |
| 28 | TVSu-600 | Nigeria | 163 | TVSu-1632 | Togo |
| 29 | TVSu-870 | Zambia | 164 | TVSu-1404 | Togo |
| 30 | TVSu-1289 | CAR | 165 | TVSu-386 | Tanzania |
| 31 | TVSu-1378 | CAR | 166 | TVSu-1851 | Zimbabwe |
| 32 | TVSu-1700 | Togo | 167 | TVSu-239 | Ghana |
| 33 | TVSu-1803 | Cameroon | 168 | TVSu-249 | Gambia |
| 34 | TVSu-101 | Mali | 169 | TVSu-295 | Burkina-Faso |
| 35 | TVSu-1892 | Botswana | 170 | TVSu-1458 | Ghana |
| 36 | MAKATA | Malawi | 171 | TVSu-1837 | Niger |
| 37 | TVSu-1480 | Ghana | 172 | TVSu-2022 | Burundi |
| 38 | TVSu-272 | Nigeria | 173 | TVSu-1362 | CAR |
| 39 | TVSu-369 | Tanzania | 174 | TVSu-1648 | Senegal |
| 40 | TVSu-671 | Nigeria | 175 | TVSu-1403 | Togo |
| 41 | TVSu-1034 | Zimbawe | 176 | TVSu-387 | Tanzania |
| 42 | TVSu-1308 | CAR | 177 | TVSu-1843 | Niger |
| 43 | TVSu-1379 | Unknown origin sourced from United Kingdom | 178 | TVSu-240 | Ghana |
| 44 | TVSu-1737 | Zambia | 179 | TVSu-250 | Gambia |
| 45 | TVSu-1813 | Cameroon | 180 | TVSu-287 | Nigeria |
| 46 | TVSu-145 | Ghana | 181 | TVSu-1464 | Ghana |
| 47 | KADZIWUNDE | Malawi | 182 | TVSu-1839 | Niger |
| 48 | TVSu-1474 | Ghana | 183 | TVSu-1667 | Senegal |
| 49 | TVSu-280 | Nigeria | 184 | TVSu-203 | Benin |
| 50 | TVSu-376 | Tanzania | 185 | TVSu-1181 | Burkina-Faso |
| 51 | TVSu-515 | Cameroon | 186 | TVSu-1295 | CAR |
| 52 | TVSu-1312 | CAR | 187 | TVSu-1165 | Burkina Faso |
| 53 | TVSu-1382 | Togo | 188 | TVSu-1101 | Zimbabwe |
| 54 | TVSu-1742 | Zambia | 189 | TVSu-1294 | CAR |
| 55 | TVSu-1821 | Cameroon | 190 | CREAM | Malawi |
| 56 | TVSu-160 | Ghana | 191 | TVSu-762 | Zambia |
| 57 | TVSu-1959 | Zimbabwe | 192 | TVSu-132 | Ghana |
| 58 | MUSANDIONE KUDA | Malawi | 193 | TVSu-99 | Mali |
| 59 | TVSu-1473 | Ghana | 194 | TVSu-1775 | Malawi |
| 60 | TVSu-289 | Benin | 195 | MASO AMARIA | Malawi |
| 61 | TVSu-379 | Tanzania | 196 | TVSu-1651 | Senegal |
| 62 | TVSu-688 | Zambia | 197 | TVSu-1164 | Burkina-Faso |
| 63 | TVSu-902 | Zambia | 198 | TVSu-1666 | Senegal |
| 64 | TVSu-1316 | CAR | 199 | TVSu-1176 | Burkina-Faso |
| 65 | TVSu-1380 | Unknown origin sourced from United Kingdom | 200 | TVSu-1186 | Burkina-Faso |
| 66 | TVSu-1756 | Malawi | 201 | TVSu-691 | Zambia |
| 67 | TVSu-1830 | Mali | 202 | TVSu-381 | Tanzania |
| 68 | TVSu-193 | Benin | 203 | TVSu-1788 | Malawi |
| 69 | TVSu-1998 | DRC | 204 | TVSu-393 | Sudan |
| 70 | CHIKOPE CHA NYANI | Malawi | 205 | TVSu-776 | Zambia |
| 71 | TVSu-290 | Benin | 206 | TVSu-984 | Zimbabwe |
| 72 | TVSu-383 | Tanzania | 207 | TVSu-1111 | Zimbabwe |
| 73 | TVSu-704 | Zambia | 208 | TVSu-1828 | Mali |
| 74 | TVSu-1146 | Guinea | 209 | TVSu-1018 | Zimbabwe |
| 75 | TVSu-1327 | CAR | 210 | TVSu-12 | Nigeria |
| 76 | TVSu-1407 | Togo | 211 | TVSu-702 | Zambia |
| 77 | TVSu-1773 | Malawi | 212 | TVSu-2012 | DRC |
| 78 | TVSu-1831 | Mali | 213 | TVSu-1301 | CAR |
| 79 | TVSu-194 | Benin | 214 | TVSu-402 | Cameroon |
| 80 | TVSu-1999 | CAR | 215 | TVSu-833 | Madagascar |
| 81 | TVSu-299 | Burkina-Faso | 216 | TVSu-939 | Zambia |
| 82 | TVSu-388 | Sudan | 217 | TVSu-1112 | Zimbabwe |
| 83 | TVSu-770 | Zambia | 218 | TVSu-1838 | Niger |
| 84 | TVSu-1147 | Guinea | 219 | TVSu-1042 | Zimbabwe |
| 85 | TVSu-1340 | CAR | 220 | TVSu-1202 | Burkina-Faso |
| 86 | TVSu-1633 | Togo | 221 | TVSu-709 | Zambia |
| 87 | TVSu-1792 | Malawi | 222 | TVSu-2014 | DRC |
| 88 | TVSu-541 | Cameroon | 223 | TVSu-1305 | CAR |
| 89 | TVSu-1922 | Senegal | 224 | TVSu-431 | Cameroon |
| 90 | TVSu-1840 | Niger | 225 | TVSu-838 | Nigeria |
| 91 | TVSu-1324 | CAR | 226 | TVSu-926 | Zambia |
| 92 | TVSu-1649 | Senegal | 227 | TVSu-1115 | Zimbabwe |
| 93 | TVSu-1419 | Togo | 228 | TVSu-1841 | Niger |
| 94 | TVSu-341 | Nigeria | 229 | TVSu-597 | Nigeria |
| 95 | TVSu-1902 | Malawi | 230 | TVSu-1056 | Zimbabwe |
| 96 | TVSu-206 | Benin | 231 | TVSu-1237 | Nigeria |
| 97 | TVSu-242 | Gambia | 232 | TVSu-723 | Zambia |
| 98 | TVSu-251 | Gambia | 233 | TVSu-2071 | Nigeria |
| 99 | TVSu-273 | Nigeria | 234 | TVSu-1306 | CAR |
| 100 | TVSu-1466 | Ghana | 235 | TVSu-441 | Cameroon |
| 101 | TVSu-1930 | Malawi | 236 | TVSu-1663 | Senegal |
| 102 | TVSu-1842 | Niger | 237 | TVSu-903 | Zambia |
| 103 | TVSu-1321 | CAR | 238 | TVSu-1139 | Guinea |
| 104 | TVSu-1653 | Senegal | 239 | TVSu-1655 | Senegal |
| 105 | TVSu-1418 | Togo | 240 | TVSu-869 | Zambia |
| 106 | TVSu-353 | Nigeria | 241 | TVSu-106 | Mali |
| 107 | TVSu-189 | Benin | 242 | TVSu-1245 | Nigeria |
| 108 | TVSu-211 | Ghana | 243 | TVSu-725 | Zambia |
| 109 | TVSu-244 | Gambia | 244 | TVSu-2074 | Nigeria |
| 110 | TVSu-252 | Gambia | 245 | TVSu-1307 | CAR |
| 111 | TVSu-254 | Nigeria | 246 | TVSu-519 | Cameroon |
| 112 | TVSu-1467 | Ghana | 247 | TVSu-1661 | Senegal |
| 113 | TVSu-1954 | Zimbabwe | 248 | TVSu-681 | Zambia |
| 114 | TVSu-1396 | Togo | 249 | TVSu-115 | Cote d'Ivoire |
| 115 | TVSu-1611 | Togo | 250 | TVSu-1251 | Nigeria |
| 116 | TVSu-901 | Zambia | 251 | TVSu-304 | Burkina Faso |
| 117 | TVSu-1417 | Togo | 252 | TVSu-1093 | Zimbabwe |
| 118 | TVSu-364 | Nigeria | 253 | TVSu-127 | Nigeria |
| 119 | TVSu-1872 | Zimbabwe | 254 | TVSu-730 | Zambia |
| 120 | TVSu-216 | Ghana | 255 | TVSu-2076 | Nigeria |
| 121 | TVSu-245 | Nigeria | 256 | TVSu-1315 | CAR |
| 122 | TVSu-303 | Burkina-Faso | 257 | TVSu-527 | Cameroon |
| 123 | TVSu-1428 | Togo | 258 | TVSu-1753 | Malawi |
| 124 | TVSu-1798 | Malawi | 259 | TVSu-677 | Zambia |
| 125 | TVSu-1964 | Swaziland | 260 | TVSu-1163 | Burkina-Faso |
| 126 | TVSu-1392 | Togo | 261 | TVSu-1182 | Burkina-Faso |
| 127 | TVSu-1617 | Togo | 262 | TVSu-335 | Nigeria |
| 128 | TVSu-90 | Mali | 263 | TVSu-1098 | Zimbabwe |
| 129 | TVSu-1414 | Togo | 264 | TVSu-1291 | CAR |
| 130 | TVSu-368 | Nigeria | 265 | TVSu-742 | Zambia |
| 131 | TVSu-1865 | Zimbabwe | 266 | TVSu-752 | Zambia |
| 132 | TVSu-217 | Nigeria | 267 | TVSu-1319 | CAR |
| 133 | TVSu-246 | Ghana | 268 | TVSu-987 | Zimbabwe |
| 134 | TVSu-302 | Burkina-Faso | 269 | TVSu-1771 | Malawi |
| 135 | TVSu-1447 | Ghana | 270 | TVSu-637 | Nigeria |
